# Falls Detection and Prevention Systems in Home Care for Older Adults: Myth or Reality?

**DOI:** 10.2196/29744

**Published:** 2021-12-09

**Authors:** Marion Pech, Helene Sauzeon, Thinhinane Yebda, Jenny Benois-Pineau, Helene Amieva

**Affiliations:** 1 Medical Research Unit 1219, Bordeaux Population Health Research Center, Inserm University of Bordeaux Bordeaux France; 2 National Institute for Research in Digital Science and Technology University of Bordeaux Bordeaux France; 3 Medical Research Unit 5800, Laboratoire Bordelais de Recherche en Informatique, National Center for Scientific Research University of Bordeaux Bordeaux France

**Keywords:** elderly people, new technologies, fall, acceptability, digital divide, aging, falls, fall prevention, detection, geriatrics, barriers, technology acceptance, home care, seniors

## Abstract

There is an exponential increase in the range of digital products and devices promoting aging in place, in particular, devices aiming at preventing or detecting falls. However, their deployment is still limited and only few studies have been carried out in population-based settings owing to the technological challenges that remain to be overcome and the barriers that are specific to the users themselves, such as the generational digital divide and acceptability factors specific to the older adult population. To date, scarce studies consider these factors. To capitalize technological progress, the future step should be to better consider these factors and to deploy, in a broader and more ecological way, these technologies designed for older adults receiving home care to assess their effectiveness in real life.

## The Era of Fall Detection and Prevention Devices for Older Adults Living at Home

These recent years have witnessed a considerable evolution of new technologies such as wearable sensors and connected applications aimed at promoting home life for older adults by providing them support in their daily activities. A frequent purpose of these technologies is the detection of falls, as falls are one of the main causes of institutionalization and functional decline [1,2]. Indeed, it has been shown that falls without severe injury multiply the risk of institutionalization by 3 while falls with severe injury multiply this risk by 10 [3]. Different types of sensors and systems for the prevention and detection of falls are currently being developed. This progress has been made possible by the development of remote data collection techniques with wireless communication technologies such as Bluetooth or Zigbee and the integration of these sensors in different contexts in research and at home, as they are smaller, less expensive, and thus more accessible to users [4].

Indeed, many wearable sensors in the Internet of Things’ paradigm have been developed with the aim of preventing and detecting falls at home [5-7]. These technologies are mostly based on monitoring and alarm systems, which are used to prevent, detect, and alert caregivers in case of fall [7]. Some provide reactive assistance to the person when a fall occurs, limiting the complications when the older adult is lying on the floor for a long time because he/she is unable to get up without help. This is typically the case of devices designed to activate an alarm when a fall occurs [8]. Other technologies such as *exergames*, *Wii Fit*, or the *Kinect* devices [9,10] act proactively by proposing preventive actions for older adults, such as home exercise programs of muscular strength and balance training. According to several studies, such home-based exercise programs could significantly reduce the risk of falls [11,12]. As a consequence, these technologies could reduce the costs and consequences of falls and increase user acceptance by providing regular information and notifications on the evolution of the user’s performance and health status, thereby encouraging older adults to use them [7].

Most tools aimed at preventing or detecting falls are based on monitoring of an individual’s motor activity by using one or several sensors [13-15]. Sensors play an essential role as they are the basic elements of data acquisition systems. These electronic devices make it possible to transform the nature of an observed physical value into an exploitable digital one. There is a huge variety of sensors: those allowing the collection of data on the physiological state of a person (eg, temperature, heart and respiratory rate, blood pressure, electrocardiogram, glycemia), those allowing the measurement of movements (eg, accelerometers, gyroscopes, magnetometers), or those detecting the geolocation of the person (eg, global positioning system). There are also ambient measurement sensors (audio and video) providing information on the environment in which the individual is. For fall detection specifically, the most frequently used measures are acceleration, angular velocity, and magnetic fields to identify body movements [13].

There are 2 types of sensors that allow the detection and prevention of falls: wearable and nonwearable ones. Wearable systems require placing sensors on the person; it may be a watch, a pendant, or a wearable camera usually attached to clothes or around the wrist [16,17]. Nonwearable systems involve sensors positioned in the person’s usual environment and use a variety of measurements such as pressure sensors [18] and ambient sensors, including visual (fixed cameras, *Kinect* sensors) [19] and acoustic (microphones) [20,21] sensors. Even though they may be perceived as more constraining for the user, wearable sensors are more effective than nonwearable ones in detecting falls because of the following reasons: first, because they can detect changes in acceleration, planes of motion, or impact with high accuracy [22]; and second, because they are not limited to a specific monitoring area in the individual’s environment [23]. To date, the most technologically and ergonomic advanced technologies are those combining several types of sensors. The data collected are multimodal (physiological, actimetric, mechanical) and thus allow more thorough analysis for both prevention and detection of falls [9,10,23-32].

Different types of connections are possible, such as wearable sensors connected to an app via a smartphone. The “SmartStep” system, for instance, uses sensors integrated into the shoe sole, which record the users’ motion. “SmartStep” is a connected electronic device, which includes a 3D accelerometer, a 3D gyroscope, pressure sensors, and Bluetooth connectivity. The system is wirelessly connected to an Android phone app, allowing both recording and visualization of data. This device has shown excellent accuracy in recognizing several daily living actions such as walking and running and has shown higher efficiency than wrist-worn devices [24,25]. Similarly, a fall detector worn in a waist belt, based on an Attitude and Heading Reference system and a barometric sensor, has been developed. This system has shown maximum sensitivity (100%) for fall detection in several studies [23,27]. Another fall detection system has been developed in an indoor environment, consisting of a belt with an accelerometer connected to a data concentrator with a wireless connection based on the Ensemble-Random Forest machine learning algorithm. This device has shown a rate of success of more than 94% for accuracy, sensitivity, and specificity in the detection of 3 types of falls (forward, backward, and sideways fall) and several actions of daily life such as walking, climbing stairs, and sitting [29]. In this line of devices integrating data from different sensors worn directly on the individual, the Bio Immersive Risk Detection System is currently being developed. It is a particularly innovative system as, in addition to the ambient, physiological, and motor sensors, the system includes a wearable camera with real-time transfer via an Android app and automatic analysis of the images to detect several risk situations, including falls and the risk of falling [30,31].

Other detection systems combine both wearable and nonwearable sensors based on the Internet of Things. For instance, there is a smart and connected home health monitoring system [26] comprising several sensors placed on household objects and sensors worn directly on the individual (belt, key ring, or pendant) with an alarm button, an interface, and software for data collection. Sensors can be attached to strategic household objects to provide information on the user’s activity or health status; for example, the pillbox (indicating adequate/inadequate medication intake) and the refrigerator door (indicating food consumption). The sensor worn by the user is used to record different movements such as walking and, especially, falling. The data processing is based on deep learning methods and hidden Markov models. The alarm button can be activated at any time by the user to alert an emergency response team. Finally, the physiological data from the different sensors are gathered on the same software platform. This system showed 99% sensitivity and 98% specificity for fall detection. Another study reported a prototype monitoring system for fall detection called “Tagcare” based on Doppler frequency recorded from a sensor worn on the person and sensors placed in the environment. The “Tagcare” system has shown high accuracy (98%) in detecting sudden movements and falls [32].

Regarding devices specifically designed for fall prevention, most are based on ambient and contextual sensors, connected to the Internet of Things, and rely on the analysis of the user’s gait and balance measures collected through different tests and physical exercises [9,10]. In a pilot study, Williams et al (2010) proposed a game console (Wii) consisting of a balance tray (like a bathroom weight scale) in which pressure sensors are integrated to monitor changes in the person’s balance, weight, and gravity while performing a recreational activity [10]. Another study reports a *Kinect* device, allowing the detection of the posture of a person with a combined system comprising a color camera coupled with an infrared emitter and its detector [28]. Although still in progress, this type of device highlights the relevance of using gait and specifically, cadence variability, while walking as predictors of falls and functional decline [28].

As may be seen, a large variety of technological solutions aiming at supporting older adults’ home life is now available and the recent results regarding fall prevention are particularly promising. Nevertheless, important challenges and barriers to a wider adoption of these devices remain [5].

## Technological Challenges

Falls refer to “the act of falling to the ground independently of one’s will. It is associated with sensory, neuromuscular, and/or osteoarticular deficiencies” [33]. Although falls in older adults are widely studied in the scientific literature, from a technical point of view, the act of falling is complex to analyze and model [34]. There are 3 types of falls: the “soft” fall, when the person holds on to a piece of furniture; the “heavy” fall, corresponding to a rapid loss of verticality associated with an impact; and the “syncopal” fall, when the person slips after losing consciousness. In addition, a distinction should be made between an effective accidental fall situation and a risk of fall. The accidental fall situation has been widely studied and its occurrence can be determined with an accuracy of 200 to 600 ms before the onset of the fall whereas the risk of falling depends on individual-specific data (physiological or environmental) and requires more sophisticated analyses.

An additional difficulty in the study of falls is that occurrence depends on the clinical context. Although falls are far less frequent in healthy individuals than in a population of frail older adults with pathological conditions, it is more difficult to detect falls in these populations. Indeed, a study from the Cambridge City over-75s Cohort on 110 older participants (over 90 years of age) considered at risk of falls equipped with an emergency call system has shown that 80% of them forgot to press the alarm button after a fall [35]. Therefore, with aging, monitoring technology solutions based on a “passive” interaction, that is, which do not require any intervention of the user, are more adapted for falls and risk of falls detection [6].

The detection systems approach has some limitations. Since falls generally follow a specific pattern (prefall, fall, and postfall) and are characterized by significant variations in movement, most approaches consider this sequence by using temporal models and by calculating the person’s movement. Many detection systems have been based on a thresholding technique, which uses a fixed threshold to detect movement variations (via wearable sensors) to distinguish falls from nonfall situations [13,22]. One of the limitations of this method is that a fixed threshold value cannot be representative of the different types of falls. Moreover, in most cases, the threshold is determined by the lowest peaks of simulated falls assessed in healthy individuals. Thus, the thresholding is quite empirical, generating numerous false positives, particularly in ecological contexts. A solution has been to turn to machine learning methods applied to measurements collected from various sensors (motion and ambient) and thus using multisensor and multimodal fusions. Using data from multiple sources ensures greater device reliability, increased robustness toward environmental interference, and improved measurement accuracy.

In addition to the difficulties inherent to fall analysis, other difficulties are related to the sensors and the Internet of Things. The first concerns the extraction of high quality and reliable data depending on both the sensors used and their sensitivities. For example, a nonoptimal placement of the sensors on the individual or on a household object would directly alter the quality of the recording or lead to errors during the reception of the signal. Connected objects are also subject to artifacts and may be interfered by the individual’s movements when they are worn on the body [36]. The second challenge concerns the collection and processing of remote data. Indeed, quality internet bandwidth cannot be ensured continuously, and the greater or lesser speed of data transmission can lead to misinterpretations and data loss [5]. Therefore, it is necessary to use backup systems and more reliable networks such as *Sigfox* to retrieve data stored in a device (eg, a smartphone) and transfer it to another device [37]. However, some information can be transmitted because there are specific conditions of security and protection of personal data. Connected devices are also limited by storage capacities and battery issues of the objects used [38,39]. Further, most technologies aiming at promoting home support are based on artificial intelligence techniques such as deep learning to proactively detect events. Deep machine learning requires a very big data volume to ensure model accuracy. Collecting such an amount of data requires a lot of time and is very costly. Finally, another potential limitation is that the data extracted from the sensors cannot be directly used by the older adult, a family caregiver, or by the clinician. Indeed, in most cases, artificial intelligence requires considerable analysis, that is, a kind of “preprocessing” so that the raw data collected by the sensors (which are data sources that did not exist before) can be transformed into meaningful, reliable, and exploitable information for the users [5]. Taken together, these limitations explain the scarce deployment of such devices in the general population or in clinical routine. Advances in digital science progressively allow finding alternatives or solutions addressing each of the technical issues previously mentioned [5]. Yet, if such technical improvements are undeniably necessary, they may not be sufficient. More research in the field of new technologies should be dedicated to social and human factors since real needs, representations, and knowledge and skills of the older adult population actually play a critical role in the effective use of the device.

## Barriers to Adopting New Technologies Among Older Adults: Between the Digital Divide and Levers of Acceptability

Despite technological advances, there are many barriers that make connected objects poorly operational for the majority of the older adult population. One of these obstacles is related to the intergenerational digital divide, which refers to an inequality in the use of and access to technology between generations, highlighting the exclusion of certain people or social groups because of their physical, social, psychological, or economic characteristics, which make them unable to access the digital world and the resources that it makes available [40]. In France, 1 out of 2 people older than 75 years does not have an internet connection at home compared to only 2% of the population in the 15-29 years age group [41]. This technological divide between the different generations may increase in the next decades owing to the exponential advance of a digitally oriented world and the nonmeeting of real needs, skills, and attitudes of older users with the opportunities provided by the current digital offer. This situation generates often stereotyped conceptions of agism in terms of interfaces, contents, and functionalities, often proving unsuitable to cover the heterogeneity of the needs and capacities of older people [42].

Another potential barrier is the social stigma generated by the exponential offer of innovative technologies (eg, home automation, fall detectors, robotics) called as “gerontechnologies” [43]. Paradoxically, the use of these new technologies to help older adults stay at home can be perceived by the general population as a new form of dependency. Indeed, in our modern societies, old age is often associated with dependence and illness. These age-related stereotypes are manifestations of “agism” with negative consequences on the mental and physical health of older adults, and as a consequence, on their access to new technologies. Biased and often stereotypical views of aging lead designers to produce solutions, which are not very accessible or inclusive for older users and contribute to the perception of older people as incompetent and unable to understand and use new technologies [44]. For example, a shared belief among the general population is that older people are not physically capable of using new technologies. However, the problem is mainly due to size (eg, small text fonts, buttons), contrast, brightness and other physical features. This problem can be solved by designing specific user interfaces for older people. Indeed, various age-related physical or sensory limitations can be counteracted with a suitable design and an optimal combination of hardware and support. Another belief is that older people lack the basic knowledge required for using new technologies. Indeed, the specific language used to describe computer objects and functions (eg, file, browser, link, desktop, download, scrollbar, cursor) is very unfamiliar to older adults. Once again, this problem could be solved by using easy and adapted language to facilitate the understanding of how the device works and how to use it [45]. Several qualitative studies using focus group methodology reveal that older adults have limited knowledge of technologies, which could be offered to them, and experience a negative stigma toward them by the simple fact that they use technological tools in their daily lives [42,46,47]. Thus, the use of technologies for home life may contribute to creating a new stereotype in the older adults who become “technologically assisted persons,” who use assistive technologies, nourishing the stigma of aging and dependence [48,49]. In turn, this vision can cause older adults to reject new technologies and thus accentuate the digital divide already prevalent in our societies [40].

Regarding “human factors” more specifically, a systematic review conducted by Hawley-Hague et al [7] reports specific intrinsic and extrinsic acceptability factors for the adoption of fall prevention and detection systems. The first intrinsic factor concerns privacy, more particularly for the systems involving automatic activation of video after a fall. To ensure the acceptability of such technologies using video recording, one solution is to use image blurring, especially in the most private areas of the home such as the bedroom or the bathroom [50,51]. Another question is whether it is appropriate to ask the older adults to set the thresholds for the activation of the video monitoring system or to turn off the video recording in the case of false alarms. At least, it should be clearly specified to the older adults what situations are likely to activate the video recording [50,52,53]. Autonomy and feeling of control may also be determining factors in the use of fall-specific technologies. To a certain extent, these technologies allow users with the loss of autonomy recovering a feeling of independence for some actions (eg, using stairs, mopping the floor in slippery areas), which are considered to be risky with advancing age and thus, regaining confidence in their functional abilities while being secured by the connected system [52,54,55]. The third factor is the perceived need by the user himself/herself for fall prevention and detection systems. This factor is influenced by the older person’s self-perceived physical, cognitive, and emotional condition, and self-esteem [48,50,51,53,54,56,57]. Faced with a society increasingly turned toward the use of new technologies, some older adults feel excluded. They fear being “overtaken,” being “out of the game,” or “unable” of appropriating and using new technology. This feeling may lead older adults to develop “technophobia,” which is an exacerbated fear of using technology and a concern about its effects on society [58,59]. In this respect, the image of one’s own aging will be an essential issue [48]. Aging persons with a positive view of themselves will be more enthusiastic about using new technology because they will perceive an opportunity to develop new skills and new experiences in their life. On the contrary, a person who has a negative image of his/her age will tend to feel “incapable” of acquiring the skills to use new technologies and will be reluctant to use it, even if their use is simplified. The life trajectory of the individual can also be a factor influencing the use of technologies and the level of anxiety associated with their use [35,48,58]. This factor refers to the experience the person has developed throughout his/her life, both personally and professionally, which will contribute to the representations of his/her own general skills acquired in this field. For example, a person who has used in his/her former occupation tools considered as “technical” may feel more armed to apprehend new technologies and may see an opportunity to capitalize his/her previous experience. This experiential factor can be favorable or unfavorable to the discovery and use of connected devices. Other factors such as anticipation of difficulties in one’s home life, the physical environment, and the type of technology may play a role in the perceived need and requirements of the technology [56]. Finally, it is important to highlight the older adults’ entourage, which is often intergenerational and often plays the role of a mediator between the technology and the older adult. In some cases, the entourage not only facilitates but also encourages, valorizes, gives meaning to the use of new technology, and provides a form of positive “social pressure,” whereas in some families where digital devices are less present and enhanced, the entourage may rather be an impeding factor [56].

Among the extrinsic factors, usability, feedback, and cost are the most important to consider in the use of fall-specific technologies [7]. Usability and usage factors refer to the individual’s perception of the object utility. This principle applies at any age of life when it is a question of appropriating a new tool of any kind [60]. The notion of utility is generally linked to a value judgment since there is no “universal” or “intrinsic” utility to an object. Similarly, the appreciation of the usefulness (or uselessness) of the object taps into individual representations, which depend on the relationship that the person has with his/her physical and social environment [61]. In the older adult population, the notion of usefulness can be linked to a specific need, for example, fighting against social isolation [62], but it is also often associated with the notion of immediacy. Indeed, the utility representation of an object depends on its capacity to address a specific and immediate need. Unfortunately, to date, very few studies consider the usefulness of technologies appreciated from the point of view of the user, in particular, when it comes to older adult users [63]. Once the tool is acquired, abandonment and poor adherence remain one of the major pitfalls [64,65]. Motivational and commitment factors depend on the ease of use of the technology, which underlines the importance of giving feedback to the user [5,61,65-67]. If the object is perceived as useful and easy to use, the person will be motivated to repeat the experience. An experience of “success” will enhance the person’s image as well as the acquired skills [66]. The connected object will not be perceived as a simple data collection system but rather as a motivational and self-engagement system [5]. Lastly, from the perspective of the older adult user, cost is an important consideration. Therefore, to guarantee a wide and egalitarian application for the whole older adult population, cost issues are very important to consider, as there is an increasing impoverishment in adults aged 65 years and older [51].

## Conclusion

This opinion paper allows drawing perspectives regarding the use of new technologies for the prevention and detection of falls among older adults and in particular, it underlines that this issue encompasses a complexity, which goes far beyond the technological challenges. Even though there is a growing interest in optimizing the accessibility of older adults to new technologies, scarce research takes into account the diversity of factors participating directly or indirectly in the digital divide and the factors of acceptability specific to the older adult population, which are decisive in the adoption of these tools. To extend this reflection, further work should consist of conducting systematic and scoping reviews addressing more specific questions by focusing, for instance, on clinical trials assessing the impact of fall detection tools and systems in frail older adults or by focusing on ergonomic studies having considered acceptability factors. To promote active and independent aging at home, it is important to encourage the use of certain assistive and preventive technologies, conveying positive messages about their benefits and ensuring that these technologies are easy-to-use, reliable, effective, and adapted to the older adults’ needs to motivate their adoption [7]. Both technological and human barriers appeal for more multidisciplinary and collaborative work between the different actors and stakeholders, that is, users, family caregivers, clinicians, and researchers from digital science, clinical sciences, and humanities who may be the key to accelerating this research.

Finally, although efforts are being made to improve the feasibility and acceptability of digital devices outside of a laboratory setting, few studies have assessed their efficacy in the “real life” of older adults selected from the general population. After the first step of development of a wide range of devices relatively accessible in terms of use and cost, evaluating such devices in large samples of older adults in ecological contexts is the second necessary step to take if we want these tools to be not just technological prototypes but operational allies really effective in promoting active aging and improving the quality of life of older adults experiencing frailty or loss of autonomy.
